# Role of fiberoptic endoscopic evaluation of swallowing (FEES) in children with suspected dysphagia

**DOI:** 10.1016/j.jped.2024.03.008

**Published:** 2024-04-26

**Authors:** Débora Bressan Pazinatto, Maria Angela Bellomo Brandão, Flávia Lima Peixoto Costa, Myrian Maria Andreotti Favaro, Rebecca Maunsell

**Affiliations:** Universidade Estadual de Campinas (UNICAMP), Campinas, SP, Brazil

**Keywords:** Deglutition disorders, Child, Endoscopy, Feeding and eating disorders, Pediatrics

## Abstract

**Objective:**

To assess FEES findings in defining oral feeding safety in children with suspected dysphagia, comparing them with clinical feeding evaluation results.

**Methods:**

This study comprised a case series involving children with suspected dysphagia, referred for evaluation by otolaryngologists and speech-language pathologists (SLPs) at a Brazilian quaternary public university hospital. These children underwent both clinical evaluations and fiberoptic endoscopic evaluation of swallowing (FEES), with a comprehensive collection of demographic and clinical data. Subsequently, the authors performed a comparative analysis of findings from both assessments.

**Results:**

Most patients successfully completed the FEES procedure (93.7%), resulting in a final number of 60 cases included in the study. The prevalence of dysphagia was confirmed in a significant 88% of these cases. Suspected aspiration on clinical SLP evaluation was present in 34 patients. Of these, FEES confirmed aspiration or penetration in 28 patients. Among the 35 patients with aspiration or penetration on FEES, 7 (20%) had no suspicion on SLP clinical assessment. All seven patients in whom clinical SLP evaluation failed to predict penetration/aspiration had neurological disorders. The median age of the children was 2.8 years, and 49 (81.6%) had neurological disorders, while 35 (58.3%) had chronic pulmonary disease. The most prevalent complaints were choking (41.6%) and sialorrhea (23.3%).

**Conclusion:**

FEES can diagnose structural anomalies of the upper aerodigestive tract and significantly contribute to the detection of aspiration and penetration in this group of patients with suspected dysphagia, identifying moderate and severe dysphagia even in cases where clinical assessment had no suspicion.

## Introduction

Swallowing disorders in children have increased and are closely related to progress in neonatal and pediatric care, which have improved the prognosis of premature infants and those with complex medical conditions.^1^, ^2^, ^3^, ^4^ Bhattacharyya et al. (2015) found that approximately 1% of children aged 3–17 reported dysphagia complaints in the previous 12 months.^5^ Of these, only 13.4% received a diagnosis, mainly for neurological disorders.^5^ Pediatric dysphagia rates are higher in at-risk populations,^5^, ^6^, ^7^ and can reach 93.8 % in children with cerebral palsy.^8^

Swallowing dysfunction in children can lead to aspiration, chronic lung disease, and poor weight gain or malnutrition. Early diagnosis is essential to prevent negative impacts.^4^ Swallowing issues stem from neuromuscular disorders, aerodigestive tract anomalies, genetic issues, and comorbidities like prematurity, cardiopulmonary, and gastrointestinal diseases.^6^ Identifying the cause is crucial for tailored treatment, often involving multiple comorbidities.^6^^,^^7^

Diagnosing dysphagia requires a high level of suspicion. Pediatricians play a key role in referring patients for specialized evaluation to rule out airway and digestive tract malformations, ensure feeding route safety, and coordinate treatment. Dysphagia demands multidisciplinary care, involving otolaryngologists, neurologists, gastroenterologists, and pulmonologists. While clinical feeding evaluation by a specialized speech-language pathologist (SLP) helps identify signs of swallowing dysfunction, it is not reliable for detecting silent aspiration.^9^

Videofluoroscopic swallow study (VFSS) and fiberoptic endoscopic evaluation of swallowing (FEES) are instruments used to evaluate swallowing function and detect aspiration in children.^7^ Although VFSS is commonly used in studies, it may not be readily available in all hospitals and is often difficult to access in public healthcare services in the country. FEES is performed in the office by the otolaryngologist and SLP or at the bedside, allowing real-time assessment of the anatomy and testing of sensibility of the pharynx, supraglottis and glottis. Anatomical variations and malformations causing dysphagia at these levels can be easily ruled out. It can also be performed in breastfeeding infants and even in children that do not feed orally with observation of swallowing of colored saliva.^1^^,^^10^ FEES has gained popularity and has been increasingly studied since its initial publication in 1988.^11^ The procedure is convenient, safe, and can provide a meaningful diagnosis.^10^, ^11^, ^12^

This study aims to assess the role of FEES findings in defining the safety of oral feeding in children with suspected dysphagia, while also comparing the clinical feeding evaluation results to FEES.

## Methods

Data were gathered from children suspected of dysphagia who underwent evaluation at a Brazilian quaternary public university hospital between May 2018 and May 2021. The study was approved by the institutional Ethics Committee (79823017.8.000.5404). Patients were referred from different pediatric outpatient clinics or from the pediatric ward or Intensive Care Unit (ICU). All patients aged from 0 to 16 years old who underwent clinical and instrumental feeding evaluation through an FEES were included. FEES and clinical feeding evaluation were performed by the same pediatric otolaryngologist and SLP, specializing in pediatric swallowing dysfunction. Children who were unable to complete FEES were excluded.

Collected data encompassed demographic and clinical details: age, gender, underlying diagnoses and co-morbidities, number of involved specialties in childcare, suspected or confirmed genetic disorders, congenital or acquired upper airway diseases, tracheostomy use, feeding route, and pulmonary-related hospitalizations in the past year. Neurological disorders included neuromuscular delay, epileptic syndromes, and cerebral palsy.

The SLP conducted a comprehensive swallowing assessment, including oral sensorimotor evaluation, static and dynamic assessment of oral structures, resting posture, control of oral secretions, and observation of feeding. Caregiver interaction and feeding methods were also evaluated. During feeding, cardiorespiratory parameters, hypotonia, breast attachment for breastfed infants, sucking pattern (lip sealing, strength, intraoral pressure, tongue movement, rhythm, and swallowing frequency), feeding time, accepted volume, removal, and approximation reactions were monitored. For non-orally fed patients, the same parameters were observed but focused on saliva swallowing.

Following clinical feeding evaluation, the SLP classified the patient as "suspected" or "not suspected" of laryngeal aspiration and proceeded to educate the caregiver about the fiberoptic endoscopic evaluation of swallowing (FEES) procedure, which was scheduled for a separate appointment. Specific findings that contributed to a classification of "suspected" aspiration included inefficient swallowing patterns characterized by weak or uncoordinated sucking and swallowing movements, the presence of intraoral residue after swallowing, coughing, choking, or signs of distress during or after feeding, abnormalities in vocal quality or changes in respiratory patterns during feeding, and a reduced ability to manage secretions or maintain adequate control of saliva.

FEES was conducted using a 3.2 mm Machida flexible fiberoptic endoscope without topical anesthesia by the same otolaryngologist while the SLP assisted with patient feeding and positioning. Caregivers sat in a clinical chair, with children on their lap, facing forward; breastfeeding positions for babies were maintained. If a nasoenteric tube (NET) was present, the endoscope passed alongside it. The assessment covered nasal cavities, pharynx, larynx, vocal cord mobility, laryngopharyngeal sensation, secretions pooling, swallow frequency, and laryngeal penetration/aspiration. Children with an oral diet were tested with stained saliva and various food consistencies. To assess children not fed orally with suspected saliva aspiration, a modified FEES was used, incorporating blue food coloring to stain saliva. This allowed for the evaluation of breastfeeding children as well. The FEES exam was considered complete when the entire planned evaluation for that specific child could be conducted. Findings were summarized and reported as positive for penetration or aspiration or negative for penetration and aspiration. Penetration was considered when secretions, food, or liquid passed within the confines of the endolarynx but did not progress below the true vocal cords. Aspiration occurred when material passed below the true vocal folds before or after swallow onset, as evidenced in the subglottic region after swallowing. Other findings such as altered sensation, early spillage, stasis, inefficient clearance of the pharynx, and oral nasal reflux were also taken into account, but only to define further therapeutic approaches.

Following the FEES procedure, the otolaryngologist and SLP conducted a collaborative interpretation of the endoscopic examination results. Dysphagia was classified into grades according to the Pediatric Dysphagia Assessment Protocol (PAD-PED): ^13^ normal swallowing, mild, moderate, or severe dysphagia.^13^ According to this classification, mild dysphagia is defined as dysphagia that can be resolved by addressing inadequacies during the feeding situation with postural, utensil, and/or flow adjustments. Moderate and severe dysphagia implies impairment of nutrition and/or hydration, and severe dysphagia indicates a high risk of aspiration, which contraindicates oral feeding.

Data on recommendations following SLP and FEES evaluations were collected, including changes in the feeding route, adjustments in consistency, restrictions on oral intake, and the implementation of enteral feeding to preserve and prevent compromise of pulmonary function. All changes were made in coordination with rehabilitative therapy.

Data were analyzed using the Statistical Package for the Social Sciences (SPSS 25.0 software). A p-value < 0.05 indicated statistical significance. Descriptive analysis included absolute and relative frequencies for qualitative variables and measures of central tendency and position for quantitative variables. The chi-square test analyzed qualitative variables, while the Mann-Whitney and Kruskal-Wallis tests compared variables between independent groups. Pairwise comparisons were adjusted using the Bonferroni correction for significant differences found in the Kruskal-Wallis test.

## Results

A total of 64 children were referred for suspected dysphagia and included during the study period. The great majority of patients completed FEES, only four (6.2%) were excluded due to failure to complete the procedure. Two patients did not tolerate the exam, and two others could not be examined because of nasal narrowing that impeded the advancement of the scope. The latter were children with syndromic craniofacial malformations.

The median age was 2.8 years (SD 3.8), comprising 32 (53.3%) males and 28 (46.6%) females. Inpatients referred from the ICU were significantly younger with a mean age of 4.3 months. Over 80 % of patients were neurologically impaired (Table 1) and there was no significant difference in age between patients with and without neurological disorders (*p* = 0.962).

**Table 1:** Patient characteristics.

	n	%	Mean/median (range)
AGE (years)			4.2/ 2.8 (0.17 –16.8)
MAIN COMPLAINT			
Choking	25	41.6	
Sialorrhea	14	23.3	
Recurrent pneumonia	11	18.3	
Difficulty gaining weight	6	10	
Vomiting	2	3.3	
Cough	2	3.3	
DURATION OF COMPLAINT (months)			26.1/ 18.0 (1.0 – 132.0)
PEDIATRIC SPECIALTY THAT REFERRED			
Gastroenterology	16	26.6	
Neurology	14	23.3	
Pneumology	9	15	
Pediatric Ward and ICU	10	16.6	
External ENT	5	8.3	
Others pediatric specialties	6	10	
Number of specialties in follow-up (beyond ENT)			3.2/ 3.0 (1.0 – 9.0)
COMORBIDITIES			
Delayed neuropsychomotor development	54	90	
Neurological disorder	49	81.6	
Chronic obstructive pulmonary disease	35	58.3	
GERD	19	31.6	
Craniofacial malformation	17	28.3	
Prematurity/ Age of birth in weeks	17	28.3	37.1/ 40 (25 - 40)
Diagnosed genetic disorder	14	23.3	
Genetic disorder under investigation	14	23.3	
Congenital heart disease	14	23.3	
Others (Autism spectrum disorder, Kidney disease)	4	6.6	
PRIMARY UPPER AIRWAY PATHOLOGIES			
Vocal cord immobility	7	11.6	
Laryngomalacia	2	3.3	
Tracheomalacia	2	3.3	

Descriptive analysis. n, absolute frequency; %, relative frequency; ENT, Otolaryngology; ICU, Intensive Care Unit; GERD, Gastroesophageal reflux disease.

The median symptom duration was 18 months, with 70% of patients experiencing symptoms for over a year (Table 1). There was no correlation between symptom duration, age, and the severity of dysphagia (p = 0.799 and p = 0.636, respectively).

All patients had already been evaluated by more than one medical specialty, with pediatric neurology being the most commonly involved (78%). The number of specialties was significantly lower in patients without evidence of dysphagia than in those with mild (*p* = 0.003), moderate (*p* = 0.016), and severe dysphagia (*p* = 0.042).

Primary upper airway pathologies were observed in over 18% of children (Table 1). Children with upper airway disease did not have more aspiration on FEES (*p* = 0.513). Moreover, three patients (5%) had undergone surgical treatment for esophageal atresia.

Nineteen patients had a tracheostomy (31.6%), and approximately 12% depended on supplemental oxygen. The average age at which tracheostomy was performed was 14 months, with a median of 9 months (range: 1–41 months). There was an association between the presence of tracheostomy and penetration or aspiration observed during FEES (p = 0.001).

The majority (55%) of patients had a history of 1–3 hospital admissions for acute respiratory events in the past year. Only 13 patients (21.6%) had not been hospitalized. Over 20% reported over 5 hospital admissions. There was no association between FEES-confirmed penetration or aspiration and number of hospitalizations in the preceding year (*p* = 0.207). Patient characterization can be seen in Table 1, Table 2.

**Table 2:** Characteristics of patients according to dysphagia classification.

Swallowing	Age*	Feeding route	Comorbidities	Main complaints	Aspiration suspected by SLP	Hospitalizations⁎⁎	Aspiration in FEES
Normal (*n* = 7)	2.4	Oral (6) GT (1)	COPD (3) GERD (3) Heart disease (2) Genetic disorder (1) Tracheostomy (1) Tracheomalacia (1) Laryngomalacia (1) Esophageal atresia(3)	Choking (4) Vomiting (1) Recurrent pneumonia (1) Cough (1)	Yes (1) No (6)	0.8	Yes (0) No (7)
Mild dysphagia (*n* = 17)	4.3	Oral (9) NET (3) GT (2) GT+oral (1) Thickened oral (1)	Neurological disorder(13) Genetic disorder (10) COPD (8) Heart disease (6) GERD (5) Tracheostomy (1)	Choking (7) Recurrent pneumonia (3) Difficulty weight gaining (3) Sialorrhea (3)	Yes (4) No (13)	1.1	Yes (0) No (17)
Moderate dysphagia (*n* = 11)	2.9	Oral (5) NET (2) GT (2) GT+oral (1) Thickened oral (1)	Neurological disorder(11) COPD (9) Genetic disorder (6) Tracheostomy (5) GERD (3) Heart disease (2)	Choking (6) Recurrent pneumonia (4) Sialorrhea(1)	Yes (9) No (2)	1.7	Yes (2) No (9)
Severe dysphagia (*n* = 25)	4.6	Oral (5) NET (2) NET+oral (2) GT (8) GT+oral (6) Thickened oral (2)	Neurological disorder (25) COPD (15) Tracheostomy (12) Genetic disorder (9) GERD (8) Heart disease (4)	Sialorrhea (10) Choking (9) Recurrent pneumonia (3) Difficulty weight gaining (2) Vomiting (1)	Yes (20) No (5)	2.5	Yes (23) No (2)

n, absolute frequency. SLP, speech language pathology; GT, gastrostomy tube; NET, nasoenteric tube; COPD, chronic obstructive pulmonary disease; GERD, gastroesophageal reflux disease; FEES, Fiberoptic endoscopic evaluation of swallowing.

⁎ Mean age in years.

⁎⁎ Mean number of hospitalizations in the last year for respiratory causes.

Dysphagia was confirmed in 88% of cases. Suspected aspiration on clinical feeding evaluation was present in 34 patients. Of these, FEES confirmed aspiration or penetration in 28 patients. Among the 35 patients with aspiration or penetration on FEES, 7 (20%) were not clinically suspected. All seven patients in whom clinical feeding evaluation failed to predict penetration/aspiration had neurological disorders. Aspiration was observed in five of these patients, while two presented with penetration. All except one of these patients were being orally fed.

The clinical feeding evaluation presented a sensitivity of 80%, specificity of 76%, positive predictive value of 82.3%, negative predictive value of 73%, and accuracy of 78.3% for detecting penetration and/or aspiration, as detailed in Table 3. There was a significant association between suspected aspiration on clinical feeding evaluation and FEES penetration (*p* < 0.001), FEES aspiration (*p* = 0.002), and the severity of dysphagia (*p* < 0.001).

**Table 3:** Distribution of patients regarding findings of SLP evaluation and FEES.

Penetration or Aspiration	FEES positive	FEES negative	N
Positive SLP evaluation	28	6	34
Negative SLP evaluation	7	19	26
n	35	25	60

FEES, Fiberoptic endoscopic evaluation of swallowing; SLP, Speech-language pathologist.

More than 50% of patients had moderate to severe dysphagia, and only 11.6% had normal swallowing (Table 4).

**Table 4:** Characteristics of feeding, clinical and instrumental feeding evaluation and proposed interventions.

	N	%
FEEDING ROUTE ON ARRIVAL		
Oral feeding	26	43.3
Modified Oral feeding	4	6.6
NET	9	15
GT	13	21.6
GT + Oral	8	13.3
ASPIRATION SUSPECTED IN THE CLINICAL FEEDING EVALUATION	34	56.6
PENETRATION IN FEES	10	16.6
ASPIRATION IN FEES	25	41.6
NO PENETRATION OR ASPIRATION IN FEES	25	41.6
SWALLOWING EVALUATION		
Normal	7	11.6
Mild dysphagia	17	28.3
Moderate dysphagia	11	18.3
Severe dysphagia	25	41.6
FEEDING CHANGES		
Thickening food	9	15
Contraindicated oral feeding:	34	56.6
Alternative feeding route (GT)	17	28.3
Exclusive GT	17	28.3
Feeding error adjustment	1	1.6
Reintroduce oral feeding	3	5

Descriptive analysis. n, absolute frequency; %, relative frequency. NET, Nasoenteric tube; GT, Gastrostomy tube.

All 25 patients with severe dysphagia had neurological disorders (Table 2), and there was a significant correlation between having a neurological disorder, aspiration on FEES (*p* = 0.002) and severity of dysphagia (*p* < 0.001). The group of patients with severe dysphagia was the only group where the main complaint was not choking but sialorrhea.

Eleven patients had no neurological disorders. Among them, 7 (63.6%) had normal swallowing, and four had mild dysphagia. Patients without neurological disorders were primarily fed orally (*p* = 0.049), with the most common complaint being choking, reported by 54.5% of them. Although three patients showed suspicion on clinical feeding evaluation, none exhibited penetration or aspiration on FEES.

Of the 60 patients evaluated, changes in feeding strategies were implemented in 56.6%, as shown in Table 4, with over 50% of children considered unsafe or oral feeding.

## Discussion

FEES was feasible and contributed to diagnosis in over 90% of patients with suspected dysphagia. The study confirmed dysphagia in most referred cases (88%), and in over half of patients (66%) either penetration or aspiration was found on FEES. Clinical feeding evaluation alone is not consistently diagnosing aspiration, as shown in the seven children with FEES-detected aspiration and/or penetration without prior suspicion. Despite the relatively high sensitivity reported for clinical feeding evaluation, one cannot underestimate the importance of FEES and/or VFSS in high-risk children.

FEES and VFSS are important for identifying swallowing issues and aspiration.^6^^,^^14^ Duncan et al. (2018) found clinical feeding evaluations unreliable for diagnosing aspiration, causing delays in diagnosis and longer wait times for confirmatory VFSS.^15^ FEES was chosen for swallowing assessment in this series due to its availability and ability to be performed alongside ENT and SLP consultations, while VFSS is not widely available in public hospitals in the region. Endoscopy offers real-time visualization of swallowing and airway protection, radiation-free testing, and saliva management assessment.^10^ Studies have validated its effectiveness in identifying swallowing abnormalities ^10^^,^^11^ and it can be repeated during follow-up as swallowing abilities may progress. In the current series, FEES feasibility was extremely high (93.7%), confirming the need for otolaryngologists in pediatrics to develop practical skills and team up with specialized SLPs. However, this study should not be viewed as evidence that FEES alone is adequate for diagnostic imaging in pediatric dysphagia in all patients. Access to VFSS should remain a priority, particularly in evaluating pediatric dysphagia in children who feed orally. Clinicians should be able to choose the most appropriate and functional examination for individualized decision-making tailored to each patient's needs.

Since VFSS was already established when FEES was introduced, the two procedures are frequently compared.^11^ Studies comparing FEES and VFSS in pediatric populations found high agreement in scoring spillage, residue, and particularly for penetration and aspiration.^16^^,^^17^ In a study of bottle-fed infants in the neonatal ICU, FEES detected more instances of penetration than VFSS, but agreement was high for aspiration (92%).^18^ According to Pavithran et al., FEES has a high specificity in detecting aspiration (82%), but a negative FEES result for aspiration should be considered in the context of aspiration risk and other endoscopic factors if VFSS is not possible.^19^ Additional research is required to establish standardized protocols for FEES in children, as highlighted by a recent systematic review examining 22 studies.^12^

In children, dysphagia is most frequently associated with multiple underlying conditions ^10^^,^^20^ that may be the cause of dysphagia or the consequence of chronic aspiration and malnutrition. Neurological disorders were highly prevalent (81.6%) in this series, exclusively associated with moderate to severe dysphagia. Among the 25 severe dysphagia patients, 12 were receiving nil by mouth but were still aspirating saliva.

Calis et al. (2008) reported a 99% prevalence of dysphagia among 166 children with cerebral palsy, positively correlated with the severity of motor impairment. Nevertheless, the infrequent reporting of feeding problems by parents illustrates how dysphagia may be underestimated.^21^ In a 10-year review, Narawane et al. (2021) found over 75% of 66 infants later diagnosed with cerebral palsy had oral or pharyngeal dysphagia, with 38% aspirating through VFSS and 64% silently.^22^ Given the high prevalence of dysphagia in children with neurological conditions, instrumental swallowing assessment is essential, as clinical feeding evaluation alone may lack sensitivity in detecting aspiration.^15^^,^^21^^,^^22^ Even when the child is not orally fed, it is important to assess if measures to control saliva aspiration are necessary. In young infants, moderate to severe dysphagia could indicate an undiagnosed neurological issue, requiring medical intervention for accurate diagnosis.

The present study revealed a mean age of 4.2 years, with 80% having three or more concurrent diagnoses. Compared to the literature, the group was first assessed for dysphagia at an older age, and it is noteworthy that the age and complexity of the population should be taken into account when comparing the present findings with other pediatric FEES cohorts. Miller et al. (2019) reported a mean age of 2.5 years in a cohort of 255 children, with 45% having three or more concurrent diagnoses in categories like neurology, cardiorespiratory, genetics, gastrointestinal, metabolic, and prematurity.^10^ This emphasizes the need for enhanced recognition and the establishment of multidisciplinary teams.

Dysphagia can lead to chronic pulmonary aspiration and respiratory issues in children,^23^, ^24^, ^25^ reflected in the high prevalence (58.3%) of patients with chronic lung disease. Aspiration pneumonia poses higher morbidity and mortality compared to community-acquired pneumonia, leading to longer and more expensive hospitalizations, increased ICU admissions, and higher 30-day readmission rates.^26^ Besides aspiration, there is growing concern regarding laryngeal penetration on FEES, as studies indicate its association with pulmonary damage and recurrent pneumonia in children.^24^^,^^25^ This is why the authors consider both penetration and aspiration as risks for oral feeding. Prioritizing safe feeding justifies modifications for moderate to severe dysphagia: adjusting food, posture, limiting oral intake, recommending gastrostomy,^14^ and surgical procedures post-FEES evaluation. These changes require concurrent rehabilitative therapy and a new clinical feeding evaluation, with FEES being valuable due to its radiation-free nature.

In this series, all eleven patients without neurological disorders had either normal swallowing or mild dysphagia, with fewer respiratory hospitalizations than those with confirmed dysphagia. Dysphagia should be considered in children with persistent respiratory issues, even without known risk factors. Lefton-Greif et al. (2006) found aspiration in 57.9% of dysphagic children with recurrent respiratory symptoms and no major comorbidities, most with liquids and silent aspiration.^27^ Investigating airway and digestive tract anomalies is crucial. Most infants recover within a year if no abnormalities are found,^28^ although dysphagia can persist for years.^27^^,^^28^ Surgical intervention, such as for laryngomalacia, laryngeal cysts, clefts, esophageal atresia, and tracheoesophageal fistula, might be needed. Our study identified tracheomalacia, laryngomalacia, and esophageal atresia, emphasizing the importance of an experienced pediatric airway specialist in the team managing dysphagic children.

The higher incidence of aspiration in children with tracheostomies cannot be solely attributed to the presence of the tracheostomy tube itself, as supported by the literature.^29^ Among the studied group, tracheostomized children had multiple risk factors, including underlying medical conditions, especially neurological disorders, and a diminished cough reflex, and some had the tracheostomy indicated for pulmonary hygiene promotion.

Older age, prolonged history of complaints, and previous involvement of multiple specialties indicate delayed referrals. Overloaded health systems may lead to long outpatient waits, but hospital admissions for acute illnesses can help identify at-risk patients for aspiration and malnutrition and conduct necessary exams and procedures. Recognizing patients with recurrent acute airway episodes, apneic spells, and feeding/swallowing issues is crucial, so pediatricians in wards and ICUs should be vigilant.

It is important to acknowledge the potential for classification bias given that the same SLP and ENT completed the clinical feeding evaluations and FEES, and no outside reviews of the reliability of findings were conducted. Despite this limitation, undoubtedly FEES remains an easily accessible, reproducible procedure that can diagnose structural anomalies of the upper aerodigestive tract and contribute significantly to the detection of aspiration and penetration in children with suspected dysphagia even when not suspected on clinical feeding evaluation. The authors recommend incorporating FEES into the diagnostic process subsequent to clinical feeding evaluation and before initiating therapeutic interventions for patients suspected of oropharyngeal dysphagia.

## Conflicts of interest

The authors declare no conflicts of interest.
